# Inflammatory and Immune-Mediated Cutaneous Diseases

**DOI:** 10.1155/2017/6793968

**Published:** 2017-12-27

**Authors:** Juarez A. S. Quaresma, Mirian N. Sotto, Anna Balato

**Affiliations:** ^1^Tropical Medicine Center, Federal University of Para, 66055-240 Belem, PA, Brazil; ^2^School of Medicine, Sao Paulo University, 01246-903 Sao Paulo, SP, Brazil; ^3^University of Naples Federico II, 80138 Naples, Italy

The concept of tissue or compartmental immunity and its importance in the development of inflammatory or infectious diseases was introduced and gained strength following publication of the works of Engwerda and Kaye [1]. From then on, we can identify peculiar characteristics of the organ-specific or tissue-specific immune responses that include the immune responses (i) at the epithelial or mucosal barrier (skin and gastrointestinal tract), (ii) in a complex organ (liver), and (ii) in organs with immunological privilege (brain and eyes) [2–5]. Skin and mucosa are examples of epithelial tissues that have a complex organization. The in situ immune system of the tissues includes both professional immune cells and cells that under certain inflammatory or infectious conditions release substances, such as antimicrobial peptides or cytokines, that interfere with the local immune response and contribute to the host's response to pro- or anti-inflammatory stimuli. These tissues function as physical barriers and in addition to cytokines, such as tumor necrosis factor (TNF), thymic stromal lymphopoietin, IL-1, IL-6, and IL-18, their immune cells secrete catecholamines, defensins, and S100. These immune cells also secrete IL-33, granulocyte-macrophage colony-stimulating factor (GM-CSF), IL-10, and chemokine ligand 27 (CCL27) and are capable of expressing Toll-like receptors (TLRs) and NACHT, LRR, and PYD domain-containing protein 3 (NLRP3), which is a component of the inflammatory process mediated by IL-1. Associated with this complex network of cytokines and components of innate immunity in the skin is a subpopulation of dendritic cells, known as Langerhans cells, that are distributed in the epidermis while in the dermis are present dendritic cells corresponding to subpopulations distinct from those seen in the epidermis, such as dermal FXIIa-positive dendrocytes, plasmacytoid dendritic cells, and CD207-positive dendritic cells. This complex network of antigen-presenting cells cooperates with the various subpopulations of lymphocytes present in the skin and with other cells, such as macrophages and NK cells, for the maintenance of local and total body homeostasis [6]. Among other immune cells, NK and NKT cells, together with the M1, M2, M4, and M17 macrophages, form a network of cells and either respond immediately to the presence and entry of harmful agents or function as effector cells through their microbicidal mechanisms. The T-cell population is heterogeneous and complex, acting in response to infectious agents in spectral infectious diseases, such as leprosy and noninfectious agents in diseases such as psoriasis and atopic dermatitis. The T-cell population comprises Th1, Th2, Th17, Th9, Th22, Th25, and Treg cells [5, 6]. Many cytokines are involved in the effector response to infectious and inflammatory harmful agents and cooperate to induce a specific regenerative environment or specific regenerative processes. In the present issue, several authors discuss the role of these immune and inflammatory factors in infectious and noninfectious skin diseases. In this respect, I. Lorthois et al. discuss the role of macrophages and neutrophils in the pathogenesis of psoriasis, calling attention to the role of innate immunity and its interrelationship with tissue microenvironmental cells as one of the contributing factors to the complex inflammatory cascade observed in the disease. Studies related to the immunopathogenesis of psoriasis involving circulating lymphocytes are discussed by J. Bartosińska et al. through the analysis of the immune response control by PD-1 expression. Later, the consequences of the systemic inflammatory response on the cardiovascular system of patients with psoriasis is discussed in the paper of S. Kaur et al. Other authors discuss the role of the complex network of inflammatory cells in the pathogenesis of urticaria and their regeneration and cutaneous involvement in inflammation-mediated diseases.

All these studies call attention to factors described recently and studied within the context of inflammatory skin diseases and their role in the evolution of these diseases. We believe that these works will give new insights into the complex dynamics of inflammatory and infectious cutaneous diseases. These concepts may serve as the basis for the development of new experimental models and may open possibilities for future investigations.

*Juarez A. S. Quaresma* *Mirian N. Sotto* *Anna Balato*
